# Supplementary material for: Detailed molecular and epigenetic characterization of the pig IPEC-J2 and chicken SL-29 cell lines
Source: iScience. 2023 Feb 20;26(3):106252. doi: 10.1016/j.isci.2023.106252 (PMC10018572; doi:10.1016/j.isci.2023.106252)
Supplement: Data S1. Complete homer output for identified motifs in Pig IPECJ-2, related to Table 2 — Homer motif analysis results for histone modifications H3K4me1, H3K4me3, H3K27ac, and enhancer elements of pig IPECJ2 cell line. P-values >1e-10 are possible false positives. Within each folder (e.g. peak_files_CTCF) are the html files showing the identified motifs when using homer (e.g. homerResults.html). [file mmc2.zip › S5/Pig_IPECJ_2/peak_files_H3K27ac/homerResults/motif10.similar.html]

motif10

## Information for motif10

C
T
G
A
C
T
A
G
A
C
T
G
A
G
T
C
G
C
T
A
T
C
A
G
G
C
T
A
C
G
A
T
G
A
T
C
G
C
A
T
A
C
G
T
A
G
C
T
A
G
C
T
  
Reverse Opposite:  

C
T
G
A
T
C
G
A
C
G
T
A
C
G
T
A
C
T
A
G
G
C
T
A
C
G
A
T
A
G
T
C
C
A
G
T
C
T
A
G
T
G
A
C
A
G
T
C
A
G
C
T
  

|  |  |
| --- | --- |
| p-value: | 1e-83 |
| log p-value: | -1.929e+02 |
| Information Content per bp: | 1.801 |
| Number of Target Sequences with motif | 61.0 |
| Percentage of Target Sequences with motif | 0.12% |
| Number of Background Sequences with motif | 1.9 |
| Percentage of Background Sequences with motif | 0.00% |
| Average Position of motif in Targets | 159.1 +/- 80.6bp |
| Average Position of motif in Background | 145.3 +/- 81.0bp |
| Strand Bias (log2 ratio + to - strand density) | 0.7 |
| Multiplicity (# of sites on avg that occur together) | 1.11 |
| Motif File: | file (matrix) reverse opposite |

### Similar de novo motifs found

|  |  |  |  |  |  |  |  |
| --- | --- | --- | --- | --- | --- | --- | --- |
| Rank | Match Score | Redundant Motif | P-value | log P-value | % of Targets | % of Background | Motif file |
| 1 | 0.634 | T C G A T C G A T G C A C A G T T A G C C G A T C T A G A T G C A T G C T G A C | 1e-66 | -153.255385 | 13.85% | 11.39% | motif file (matrix) |
